# Adenosine-Based Modulation of Brain Activity

**DOI:** 10.2174/157015909789152173

**Published:** 2009-09

**Authors:** DETLEV BOISON

The purine ribonucleoside adenosine is an endogenous modulator of brain function uniquely positioned to integrate a wide variety of signalling pathways. Due to its early evolutionary roots – adenosine was already part of the “RNA-world” and strategically placed to bridge cellular activities (as building block of nucleic acids) with energy supplies (adenosine-5’-triphosphate, ATP) – it is not surprising that adenosine has evolved into a “master regulator” with important functions in every known form of life and in almost every organ system studied. This Hot Topics Issue is devoted to the role of adenosine as regulator of brain activity in health and disease. Due to the advent of modern molecular tools, the adenosine-field has experienced a revival during the past decade that led to the development of experimental adenosine-based therapeutics. Therapeutic modulation of the adenosine system holds much promise for the soft therapy of a wide variety of neurological or neuropsychiatric conditions. However, the ubiquitous distribution of components of the adenosine-based regulatory system constitutes a therapeutic challenge that needs to be met by specificity.

**	Bruno Frenguelli **and **Nicholas Dale **provide an introduction to the topic and discuss the potential sources of adenosine and ATP, and mechanisms of their release into the extracellular space. With microelectrode biosensors that are specific for the online detection of adenosine or ATP *in vitro* or *in vivo* the authors have developed and used an innovative tool to study changes in ambient adenosine as a consequence of ischemia and epilepsy in real time. **Ana Sebastiao **and **Alexandre Ribeiro **discuss the downstream pathways of adenosine and how adenosine can tune and fine-tune the activity of a wide spectrum of neurotransmitters and neuromodulators. A high degree of complexity becomes possible via receptor-receptor interactions and crosstalk between adenosine receptors and transporters. While the first two chapters focus on molecular mechanisms, with an emphasize on upstream and downstream pathways, respectively, the remaining review articles are devoted to the role of adenosine regulation and dysregulation within the context of specific physiological and pathophysiological conditions. **Hai-Ying Shen** and **Jiang-Fan Chen** present novel psychopharmacological findings that highlight the unique role of the adenosine A_2A_ receptor as a key player for neuropsychiatric conditions including addiction, mood-control, cognitive function, and schizophrenia. The molecular basis for these interactions has been elaborated in transgenic mice with brain-region specific deletions of the A_2A_ receptor (e.g. striatum *versus* forebrain); in particular, the A_2A_ receptor emerges as a crucial upstream regulator of both glutamatergic and dopaminergic neurotransmission. **Anisur Rahman** highlights current knowledge on the role of the adenosine system in Alzheimer’s disease. The beneficial effects of caffeine – a non-selective adenosine receptor antagonist – and of specific A_2A_ receptor antagonists are discussed. **Rebecca Williams-Karnesky **and **Mary Stenzel-Poore **highlight the role of adenosine as endogenous neuroprotectant of the brain within the context of stroke. Reprogramming of the brain and immune responses to adenosine signaling may be an underlying principle of tolerance to cerebral ischemia.  Insight into the role of adenosine in various preconditioning paradigms may afford insight into new uses for adenosine as both an acute and prophylactic neuroprotectant. **Theresa Lusardi **discusses current knowledge about the role of adenosinergic signalling in traumatic brain injury (TBI). In the central nervous system, adenosine (dys)regulation has been demonstrated following TBI, and correlated to several TBI pathologies, including impaired cerebral hemodynamics, anaerobic metabolism, and inflammation. In addition to acute pathologies, adenosine function has been implicated in TBI comorbidities, such as cognitive deficits, psychiatric function, and post-traumatic epilepsy. **Theresa Bjornes **and **Robert Greene **discuss the role of adenosine in sleep with a focus on slow wave sleep. This review highlights how adenosine influences sleep, and how sleep influences the adenosine system. New molecular data give a wealth of information, which receptors and mechanisms contribute to sleep control. **Srdjan Vlajkovic, Gary Housley, **and **Peter Thorne **present new insights how the adenosine system is involved in the regulation and maintenance of auditory functions. An interest in a potential otoprotective role for adenosine has recently evolved, fuelled by the capacity of A_1_ adenosine receptors to prevent cochlear injury caused by acoustic trauma and ototoxic drugs. The balance between A_1 _and A_2A_ receptors is conceived as critical for cochlear response to oxidative stress, which is an underlying mechanism of the most common inner ear pathologies. Novel therapeutic targets and strategies that are based on modulation of adenosine signalling are presented. Finally, **Susan Masino **and colleagues discuss the potential of a ketogenic diet to raise brain levels of adenosine with a focus on exploiting the therapeutic potential of adenosine augmentation in epilepsy. As a closing statement for this special issue she presents the rationale how a ketogenic diet – or adenosine augmentation in general – could be of therapeutic benefit for brain injury, inflammatory and neuropathic pain, autism, and hyperdopaminergic disorders.  

This Hot Topics Issue is expected to provide an overview on the rapidly emerging field of adenosine function and dysfunction in neurological and neuropsychiatric conditions with a focus on molecular mechanisms and possible therapeutic interventions. It can be expected that adenosine-based therapeutics for a wide variety of disorders will further be developed and refined during the next decade. This Hot Topics Issue shall provide an impetus for further research in this emerging area.

